# Assessment of Bone Invasion and Its Correlation With Brandwein-Gensler Criteria in Oral Squamous Cell Carcinoma

**DOI:** 10.7759/cureus.61194

**Published:** 2024-05-27

**Authors:** Sakshi Akolkar, Alka Hande, Swati K Patil, Archana M Sonone, Aayushi Pakhale

**Affiliations:** 1 Department of Oral Pathology and Microbiology, Sharad Pawar Dental College and Hospital, Datta Meghe Institute of Higher Education and Research, Wardha, IND

**Keywords:** lhr, wpoi, pni, lymphocytic host response, perineural invasio, worst pattern of invasion, brandwein-gensler criteria, oscc

## Abstract

Background

The most prevalent form of head-neck cancer is squamous cell carcinoma (SCC). Apart from all sites like the tongue, labial mucosa, and buccal mucosa, the prevalence of oral squamous cell carcinoma (OSCC) is more common in gingivobuccal sulcus due to the habit of keeping tobacco quid. With regards to anatomical relationships in the mouth and proximity to bone, OSCC invades the maxilla and mandible. However, bone invasion significantly influences the pathological staging of OSCC. Histological parameters such as Brandwein-Gensler worst pattern of invasion (WPOI), lymphocytic host response (LHR), and perineural invasion (PNI) hold significance for determining the need for adjuvant therapy. This study aims to correlate Brandwein-Gensler Criteria (BGC) with bone invasion and also to include the bone invasion criteria as a prognostic parameter in OSCC. This study aimed to assess bone invasion and correlate it with Brandwein-Gensler criteria in OSCC.

Methods

The research was conducted retrospectively, analyzing 65 cases of OSCC that underwent surgical intervention. Data was gathered from the Oral Pathology department's archives at Sharad Pawar Dental College (SPDC), Wardha. Pathologists assessed bone invasion without the knowledge of other factors to minimize bias. Subsequently, the cases were classified into well-differentiated (WDSCC), moderately differentiated (MDSCC), and poorly differentiated squamous cell carcinomas (PDSCC) based on histological grading, followed by the evaluation of WPOI, LHR, and PNI using the Brandwein-Gensler risk scoring system.

Results

This study found a notable association between bone invasion and BGC, with a calculated significance level of p = 0.047. LHR shows patterns as 1, 2, and 3. There were five (7.6%) cases with pattern III, 45 (69.23%) cases with pattern II, and 15 (23.08%) cases with pattern I. Similarly, PNI is scored as 0, 1, and 3. There were seven (10.77%) cases with score 3, 17 (26.15%) with score 1, and 41 (63.03%) with score 0. In the case of the WOPI, which is classified as patterns I to V, there were seven (10.77%) cases with pattern V, 27 (41.54%) cases with pattern IV, 23 (35.38%) cases with pattern III, and eight (12.231%) cases with pattern II, whereas no cases were noted with pattern I.

Conclusion

Although bone invasion and BGC are independent parameters, the BGC score should be considered in treatment planning. Patients with bone invasion and those with a higher BGC score should be strongly considered for adjuvant treatment.

## Introduction

Cancer refers to the unchecked proliferation of cells that infiltrate and damage nearby tissues. Oral cancer manifests as a tiny, unfamiliar, and inexplicable growth or ulceration in various parts of the mouth, such as the lips, buccal mucosa, sinuses, tongue, hard and soft palate, and the area extending up to the oropharynx [1]. Cancers affecting the oral cavity and pharynx rank as the sixth most prevalent type of cancer globally [2]. Oral squamous cell carcinoma (OSCC), the most common malignancy of the head-neck region, has drawn the attention of oral and maxillofacial specialists. This is due to its high risk and mortality rates in many countries, in addition to the intricate social and economic ramifications it has for individuals who manage to survive this incredibly debilitating condition [3]. In India, the illness ranks third among females and is the most frequent malignancy in males [2]. Oral malignancies are commonly known as OSCC and have been linked to smoking, alcohol drinking, and betel nut chewing [4]. The major risk factors for malignancies include the consumption of alcohol, tobacco, and betal quid and human papillomavirus (HPV). Other factors that contribute to its development include other bacteria, dietary factors, vitamins and mineral deficiencies, immune system function, exposure to toxins in the environment, work-related exposures, and inherited abnormalities [5]. Sustained infections, such as HPV, are one of the hazards related to the development of oral malignancies. Lack of awareness, exposure to risky climatic circumstances, and behavioural risks are all markers of an extensive range in worldwide prevalence [1].

OSCCs are malignant tumours that typically infiltrate the mandibular bone, making the invasion of bone a prevalent clinical issue. OSCCs infiltrate the lower jaw bone in an erosive, mixed, or infiltrative manner, which corresponds to clinical behaviours [6]. Surgery is the first step in treatment, and then radio-chemotherapy or neoadjuvant therapy may come next. This has a big impact on the function, appearance, and quality of life of the patients. Because of their close proximity to the bone, malignant cells can enter bone structures very easily. Malignancies of the tongue, retromolar trigone (RMT) region, and floor of the mouth invade the lower jaw in 42%, 48%, and 62%, respectively. Regardless of extent, site, or kind of invasion of bone, malignant tumours are classified as T4 by the Union for International Cancer Control's (UICC) TNM (tumour (T), extent of spread to lymph nodes (N), and presence of metastasis (M)) classification system. Generally, there are two types of patterns that can be identified through which invasion takes place in bone: an erosive pattern, which is shown histologically as a tumour mass that splits away from a bone by a connective tissue [7]. The American Joint Committee on Cancer (AJCC) staging classification for OSCC continues to define malignancies that invade through cortical bone as T4, which automatically assigns the patient to stage IV illness with prognostic and treatment consequences. This is true even though recent research indicates that, when puzzling variables like tumour size and involved surgical margins are taken into consideration, invasion of bone is not an independent prognostic predictor [8]. It is common for OSCCs to pierce the mandible [9]. Bone invasion is a key prognostic feature in late-stage OSCC, linked to poor quality of life and decreased life expectancy [10].

Indeed, bone invasion significantly influences the pathological staging of OSCC. In addition to pathological staging, histopathological parameters are crucial in determining the need for adjuvant radiotherapy. To study these parameters, Brandwein-Gensler proposed a risk model that incorporates factors such as lymphocytic host response (LHR), perineural invasion (PNI), and Brandwein-Gensler worst pattern of invasion (WPOI). These elements provide valuable insights into treatment planning and prognostic assessment in OSCC cases.

## Materials and methods

The study was carried out in a retrospective manner and included 65 surgically operated cases of OSCC. The data was retrieved from the archives of the Department of Oral Pathology and Microbiology, Sharad Pawar Dental College (SPDC), Wardha. The invasion of bone was assessed by the pathologist in a blinded manner in order to eliminate bias. Based on the degree of differentiation, the histological grading of the OSCC patients was divided into well-, moderately, and poorly differentiated carcinomas. Brandwein-Gensler proposed a novel multiparameter histologic ‘‘risk assessment’’ model that predicted local recurrence and overall survival, which included WPOI, LHR, and PNI. The WPOI is classified as patterns I, II, III, IV, and V and accordingly the scores are assigned. The LHR is classified into patterns I, II, and III respectively corresponding to strong, intermediate, and weak. Similarly, PNI is classified and scores are assigned (Table 1). 

**Table 1:** Scoring system for assessing risk (Revised from Brandwein-Gensler et al., 2010) [11] WPOI: Worst pattern of invasion LHR: Lymphocytic host response PNI: Perineural invasion

Variables		Description	Point assigned
WPOI	Pattern I	Pushing border (advancing boundaries)	0
	Pattern II	Finger-like growth (digitate expansion)	0
	Pattern III	Significant isolated clusters, each comprising more than 15 cells	0
	Pattern IV	Tiny tumor clusters, containing 15 cells or less per cluster	+1
	Pattern V	Secondary tumour nodules, measuring at least 1mm apart from the primary tumour (under 20x magnification) or the nearest adjacent satellite	+3
LHR	Pattern I strong	A compact and comprehensive immune response surrounding tumour lymphoid nodules at the leading edge in every 4x field	0
	Pattern II intermediate	Moderate host response, with lymphoid nodules present in some, but not all, 4x fields	+ 1
	Pattern III weak	Minimal or absent host response, lacking lymphoid nodules	+ 3
PNI	None	None	0
	Small nerves	Tumor encircling nerves, with a diameter of less than 1mm.	+ 1
	Large nerves	Tumor enveloping nerves, measuring equal to or exceeding 1mm in diameter under 20x magnification.	+ 3

Furthermore, these scores are summed up and the neoadjuvant therapy is decided (Table 2).

**Table 2:** Risk score and neoadjuvant treatment recommendations [12]

Total Risk Score (sum of all point allocations)	Risk of local recurrence	Chance of survival	Recommendations for neoadjuvant treatment
0	Low	Good	Neoadjuvant radiotherapy showed no benefit in local disease-free outcomes
1 to 2	Intermediate	Intermediate	Neoadjuvant radiotherapy demonstrated no advantage in local disease-free progression
3 to 9	High	Poor	Radiotherapy recommended irrespective of 5 mm margins

## Results

In the present study, value of significance was set to 0.05 (p =0.05). Thus, bone invasion has been tabulated against Brandwein-Gensler criteria (BGC). A significant correlation was observed between bone invasion and BGC. A bone invasion with a BGC value of p = 0.047 was seen.

The study enrolled a total of 65 cases, predominantly male (84.62%), with ages ranging from 25 to 75 years and a mean age of 50 years at diagnosis. The most common primary tumour sites were the floor of the mouth and retromolar trigone. Lymph node metastasis was detected in 34 (52.30%) patients, with extracapsular spread observed in 18 (27.69%) individuals. Tumour size varied from 1.5 to 12 cm, with depths ranging from 0.7 to 7 cm. Among the tumours, 19 were well-differentiated (29.69%), while 46 were moderately differentiated (70.77%), and there were no cases of poorly differentiated tumours. Histopathologically, the majority of cases exhibited MDSCC with deep infiltration. Neoplastic invasion commonly affects blood vessels, connective tissue, nerves, salivary glands, and bone structures.

**Figure 1:**
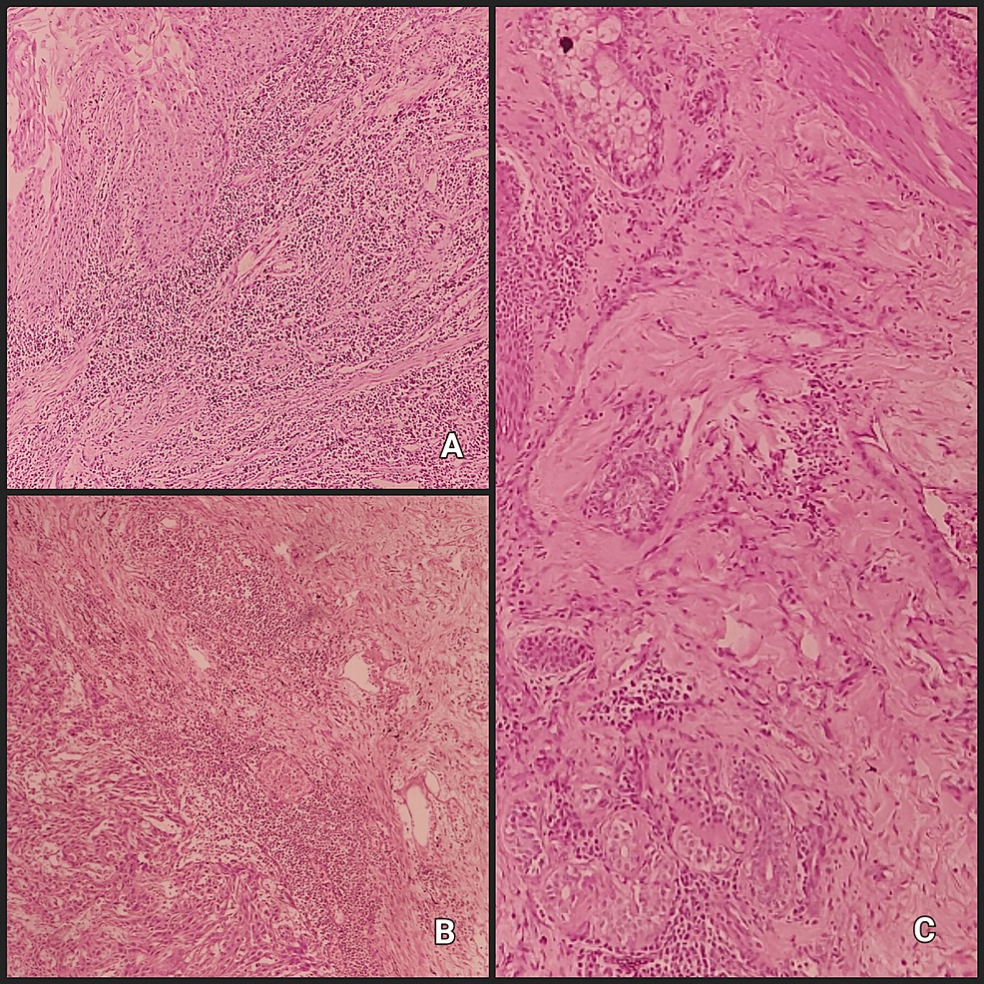
Lymphocytic host response: (A) Pattern I: A compact and comprehensive immune response surrounding tumor lymphoid nodules at the leading edge in every 4x field, (B) pattern II moderate host response, with lymphoid nodules present in some, but not all, 4x fields, (C) pattern III minimal or absent host response, lacking lymphoid nodules.

Lymphocytic host response shows patterns as 1, 2, and 3. There were five (7.6%) cases with pattern III, 45 (69.23%) cases with pattern II, and 15 (23.08%) cases with pattern I (Table 3 and Figure 1).

**Table 3:** Lymphocytic host response

Pattern	Cases	Percentage (%)
I	15	23.08
II	45	69.23
III	5	7.69

 

**Figure 2:**
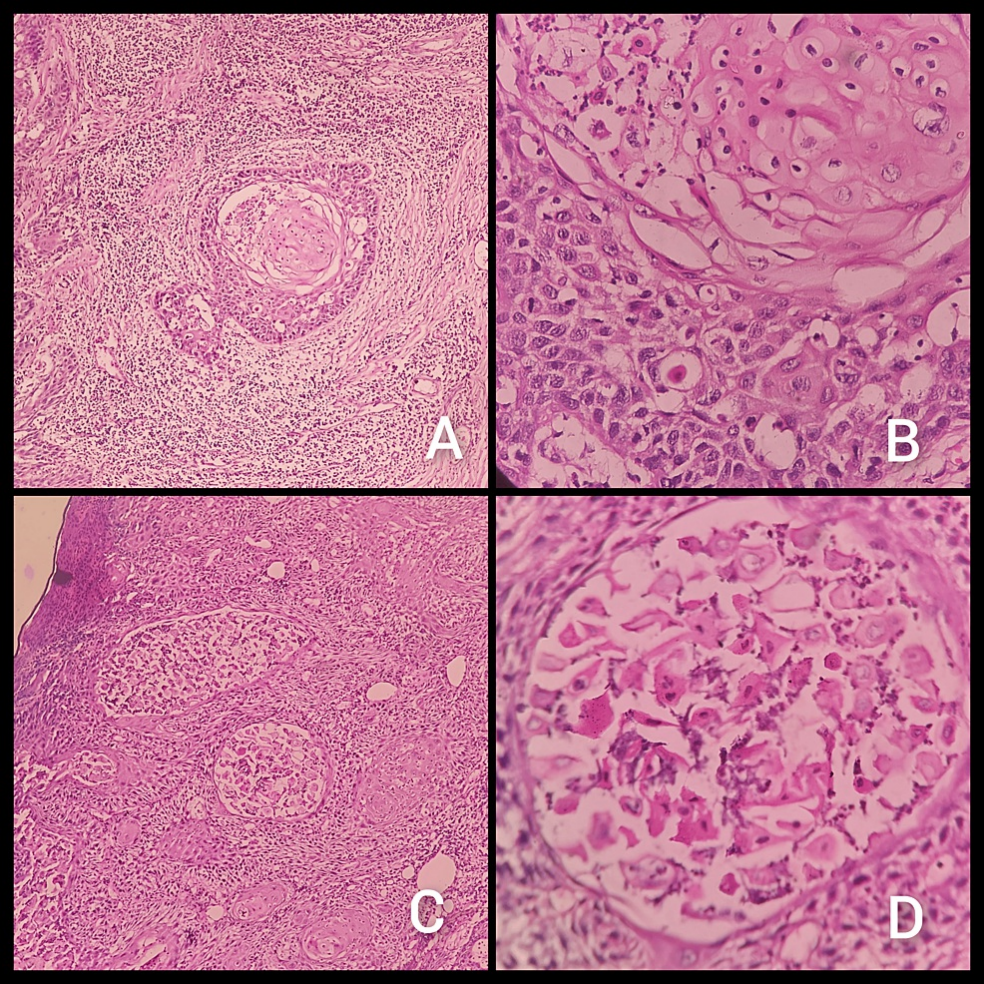
Perineural invasion. Tumor involving epineurium: (A) 10x and (B) 40x. Tumor involving endoneurium, perineurium and epineurium: (C) 10x (D) 40x.

Similarly, PNI is scored as 0, 1, and 3. There were seven (10.77%) cases with score 3, 17 (26.15%) with score 1, and 41 (63.03%) with score 0 (Table 4 and Figure 2).

**Table 4:** Perineural invasion

Score	Cases	Percentage (%)
0	41	63.08
+1	17	26.15
+3	7	10.77

 

**Figure 3:**
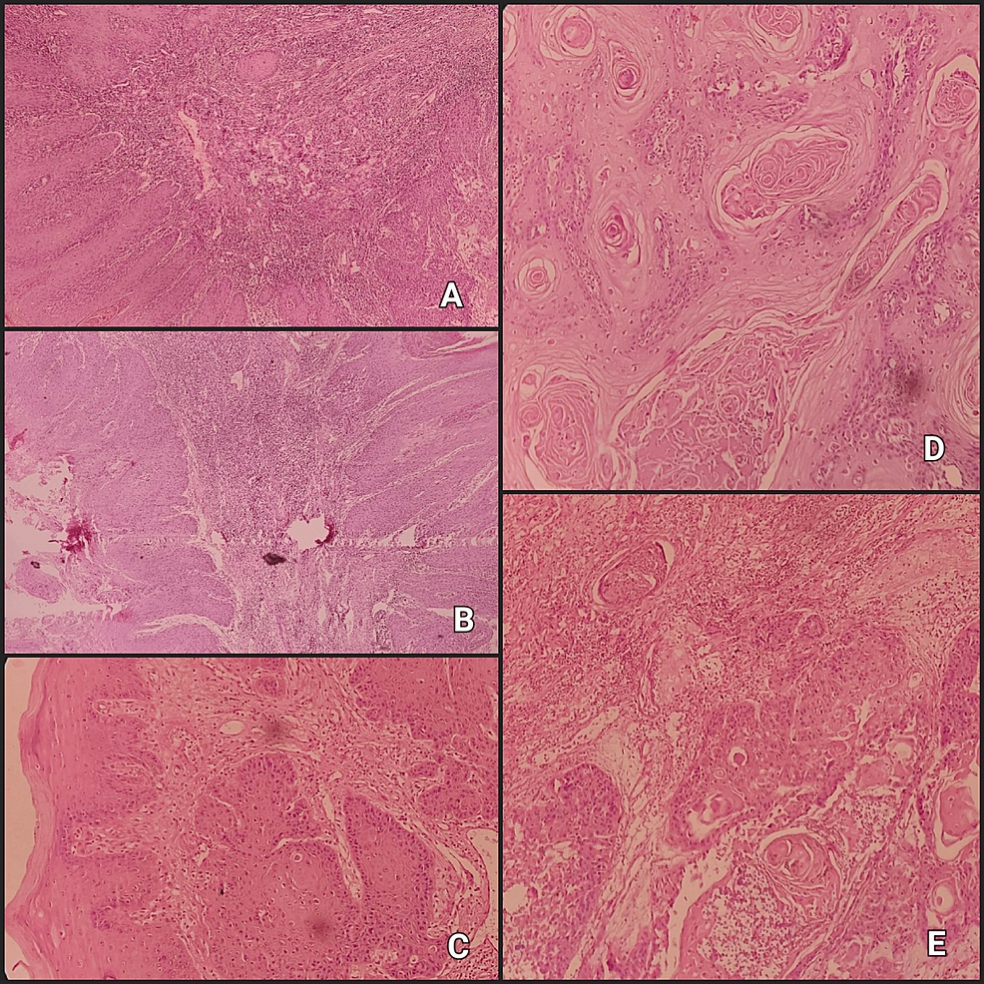
Worst pattern of invasion: (A) Pattern I pushing border (advancing boundaries), (B) pattern II finger-like growth (digitate expansion), (C) pattern III significant isolated clusters, each comprising more than 15 cells, (D) pattern IV tiny tumour clusters, containing 15 cells or less per cluster, and (E) pattern V secondary tumour nodules, measuring at least 1 mm apart from the primary tumor (under 20x magnification) or the nearest adjacent satellite.

In the case of the WPOI, which is classified as patterns I to V, there were seven (10.77%) cases with pattern V, 27 (41.54%) cases with pattern IV, 23 (35.38%) cases with pattern III, and eight (12.231%) cases with pattern II, whereas no cases were noted with pattern I (Table 5 and Figure 3).

**Table 5:** Worst pattern of invasion

Pattern	Cases	Percentage
I	0	0%
II	8	12.31%
III	23	35.38%
IV	27	41.54%
V	7	10.77%

 

It was observed that a positive bone invasion was seen in 18 (27.69%) cases out of 65 (Table 6).

**Table 6:** Bone invasion

	BGC			
	0	1 to 2	3 to 9	Total
Positive	0	9	9	18
Negative	11	23	13	47
Total	11	32	22	65

**Figure 4:**
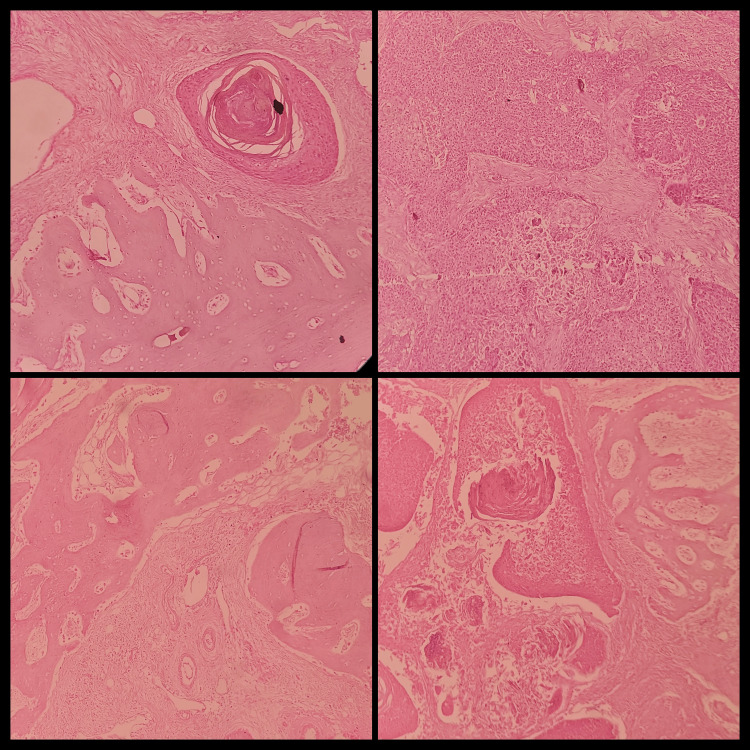
Bone invasion: Invasive growth pattern seen and the associated changes in the stroma are also shown.

Some histopathological aspects of OSCC cases with bone invasion are depicted in Figure 4.

## Discussion

India faces a significant burden of OSCC, with an estimated 75,000-80,000 new cases reported annually. While early-stage oral cancer has a more favourable prognosis, a notable portion (12%-14%) of patients experience relapse, which leads to locoregional or distant recurrences. Patients had a final histological evaluation by a trained pathologist who classified them using the Brandwein-Gensler (BG) model risk classifications in order to assess the risk of recurrence. Three key components are included in this model: WPOI, PNI, and LHR. The approach of Brandwein-Gensler et al. is used to evaluate each of these components. The patients are categorized as low, moderate, or high-risk based on their overall assessments. The BG risk model can be a useful tool for predicting loco-regional recurrences in OSCC patients receiving adjuvant therapy after primary surgery [13]. Under a microscope, the LHR at the margin of the tumour is examined using light microscopy. Based on the existence of lymphoid nodules, dense populations of lymphocytes along the tumour-host interface can be categorized as strong, intermediate, or limited. In the 10x microscopic field, tumours with a strong LHR will have at least one lymphoid nodule at the tumour interface; tumours below this threshold will have one or more lymphoid nodules [14]. A higher point value is assigned to carcinomas that spread beyond the main mass (worst POI (WPOI) type 5, which is assigned three points) compared to carcinomas that form small islands and stay close to the main tumor (WPOI type 4, which is assigned one point). The risk model is nonlinear, grouping together nonaggressive patterns of invasion. Compared to WPOI type 4, WPOI type 5 is distinct to our model, present in 25% of this cohort, and substantially related to worse outcomes [11]. Three layers make up nerve sheaths: the outermost layer, called the epineurium, is a dense layer of connective tissue that encases nerves; the innermost layer, called the perineurium, envelops nerve fascicles and individual axons as well as Schwann cells.

PNI is described histopathologically as malignant cells that migrate through, into, or around a nerve. More specifically, PNI is defined as tumour involvement that occupies any one of the three nerve sheath layers or at least 33% of the nerve's diameter [15]. PNI accounts for over 80% of head and neck cancer cases. PNI is frequently associated with neurotropic cancers. It occurs when malignant cells migrate towards nerve bundles in the tissue. Unlike lymphovascular invasion, PNI involves the spread of tumour cells along the nerve pathways, leading to symptoms such as pain and the dissemination of the tumour to distant areas [16]. Research has shown that 42% of cases of tongue OSCC, 48% of cases in the RMT region, and 62% of cases of oral floor malignancies have lower jaw invasion of bone [17]. The invading OSCC into the adjacent upper and lower jaw bones can contribute to the staging of the tumour. The diagnosis of the invasion of bone typically involves imaging techniques such as CT scans and MRI. Treatment often involves bone resection. OSCC bone invasion is classified into three types: erosive, infiltrative, and mixed [18]. The extension of OSCC into the mandible is indicative of advanced disease and typically classifies the tumor as stage IV. This involvement of the mandible is often associated with a poor prognosis for the patient [19].

The observation of our study in relation to parameter of Brandwein-Gensler like LHR, PNI, and WPOI are as follows: LHR shows patterns as 1, 2, and 3. We observed that there were five (7.6%) cases with pattern III, 45 (69.23%) cases with pattern II, and 15 (23.08%) cases with pattern I. Similarly, PNI is scored as 0, 1, and 3. There were seven (10.77%) cases with score 3, 17 (26.15%) with score 1, and 41 (63.03%) with score 0. Also, in the case of WPOI, which is classified as I to V, there were seven (10.77%) cases with pattern V, 27 (41.54%) cases with pattern IV, 23 (35.38%) cases with pattern III, and eight (12.231%) cases with pattern II, whereas no cases were noted with pattern I.

Further, with respect to bone invasion, there were 18 (27.69%) cases with positive bone invasion, compared to 47 (72.30%) without positive bone invasion. In our study, a total of 65 cases of OSCC were included, among whom 18 exhibited positive bone invasion. According to the criteria set by Brandwein-Gensler, patients with a score ranging from 3 to 9 should strongly consider adjuvant radiotherapy, regardless of whether bone invasion is present or not. Therefore, out of the total 65 patients, a total of 22 patients (18 with positive bone invasion and additional patients with scores of 3-9) should opt for adjuvant radiotherapy. This is proved by the statistical analysis, which showed a significant correlation between bone invasion and Brandwein-Gensler criteria with a p value of 0.047.

As per literature, there are no studies showing the correlation of BGC with bone invasion. However, the degree of invasion of bone as a prognostic factor in OSCC has been evaluated. Ebrahimi et al. assessed the degree of invasion of bone as a separate prognostic factor in OSCC. 102 patients with verified bone invasion were among the 498 OSCC patients who underwent curative surgery and were included in the retrospective review. Invasion by bone was classified as cortical or medullary, as per their presence. Further, its relationship to disease control and survival was investigated. Following multivariate analysis to account for the relevant confounders, their results showed no significant correlation between cortical invasion and either overall (P value as 0.48) or disease-specific (P value as 0.63) survival. On the other hand, medullary invasion has been identified as a reliable indicator of decreased overall and disease-specific survival. This decrease in survival appeared to be mainly related to a higher chance of distant metastatic failure (P value =0.037) as opposed to a local (P value = 0.51) or regional (P value = 0.14) recurrence. Furthermore, tumour size substantially affected survival within the sample of patients with medullary invasion (P =0.029) [8]. In a study led by Cassie Fives et al., the significance of bone invasion in oral cancers has been investigated. They found bone invasion in 31 cases (32%), all of which demonstrated medullary involvement upon pathology slide review. For all the cases, the median follow-up was 36 months; for those who did not die from cancer, it was 53 months. Bone invasion was strongly linked with poorer overall survival (OS) (P = 5.0005) and local control (LC) (P = 5.04) in tumours 4 cm or larger. Furthermore, findings from the entire cohort showed that medullary invasion, postoperative radiotherapy, and positive pathologic nodal status were independent predictors of poorer overall survival. Regardless of the small size of the initial tumour, their result emphasizes that mandibular medullary invasion of bone is a poor prognostic indication in oral malignancies. They support thinking about postoperative radiation in these kinds of situations [9].

## Conclusions

Although bone invasion and BGC are independent parameters, we must consider the BGC score in treatment planning, as cases with a 3-9 score require adjuvant treatment. Patients with bone invasion and those with a higher BGC score should be strongly considered for adjuvant treatment.
